# The Rice Qa-SNAREs in SYP13 Subfamily Are Involved in Regulating Arbuscular Mycorrhizal Symbiosis and Seed Fertility

**DOI:** 10.3389/fpls.2022.898286

**Published:** 2022-05-18

**Authors:** Ying-Na Liu, Cheng-Chen Liu, Rui Guo, Li Tian, Jian-Fei Cheng, Ya-Nan Wu, Dong Wang, Bin Wang

**Affiliations:** ^1^Department of Biology, School of Life Sciences, Nanjing University, Nanjing, China; ^2^Department of Biochemistry and Molecular Biology, University of Massachusetts Amherst, Amherst, MA, United States

**Keywords:** rice (*Oryza sativa*), arbuscular mycorrhiza (AM), SNARE, *OsSYP13*, functional redundancy

## Abstract

Qa-SNARE gene *SYP132* (isoform α) was previously reported to affect arbuscular mycorrhizal (AM) symbiosis in the legume species *Medicago truncatula*. In non-legumes especially monocots, it remains unknown whether certain *SNARE* genes are also involved in AM symbiosis. In this work, we studied a rice orthologous gene *OsSYP132*, which showed induced expression in mycorrhizal roots and two paralogous genes *OsSYP131a* and *OsSYP131b*, which were not induced by the AM fungus *Rhizophagus irregularis*. After employing CRISPR/Cas9 technique to generate their mutants, the *Ossyp131a* homozygous mutant T_0_ plants exhibited a dwarf phenotype and produced no fertile seeds, indicating a required role of this gene in seed fertility. Unlike the case in legume, the *Ossyp132* mutants exhibited normal mycorrhizal phenotype, so did the *Ossyp131b* mutants. In the *Ossyp131b Ossyp132* double mutants, however, the colonization rate and arbuscule abundance level decreased markedly, indicating an impaired fungal proliferation ability in rice roots. Such a defect was further confirmed by the reduced expression levels of AM marker genes. Our results in rice therefore demonstrated that while SYP13II members showed evolutionary and induction patterns specific to symbiosis, AM symbiosis is in fact controlled by the combined action of both SYP13I and SYP13II clades, revealing a functional redundancy among SYNTAXIN genes in mutualism.

## Introduction

Arbuscular mycorrhiza (AM) symbiosis is a mutualistic endosymbiosis formed between most terrestrial plants and Glomeromycotina fungi (Wang and Qiu, 2006; Smith and Read, 2008; Spatafora et al., 2016). During the symbiosis, the fungal hyphae penetrate root inner cortical cells and develop highly branched, bush-like structures called arbuscules (Genre et al., 2008). Arbuscules are surrounded by the peri-arbuscular membrane (PAM), which is continuous to the cell plasma membrane. Through the interface of PAM and arbuscules, nutrient exchanges occur between symbiotic partners, where plants provide carbohydrates, mainly in the form of fatty acids, to fungi and fungi supply mineral nutrients, especially phosphorus and nitrogen, to plants (Smith and Smith, 2011; Bravo et al., 2017; Jiang et al., 2017; Keymer et al., 2017; Luginbuehl et al., 2017; Wang et al., 2017, 2020; Wipf et al., 2019).

In eukaryotic cells, exocytosis via membrane trafficking is a fundamental process involving the fusion of secretory vesicles with target membranes (Kanazawa and Ueda, 2017). It is mediated by a group of SNARE (soluble *N*-ethylmaleimide-sensitive factor attachment protein receptor) proteins that are divided into two classes, Q (glutamine)-SNAREs and R (arginine)-SNAREs (Fasshauer et al., 1998). When secretory vesicles move close to the target membrane, one R-SNARE located on the vesicle membrane (also called v-SNARE) and three types of Q-SNAREs (Qa, Qb, and Qc) on the target membrane (also called t-SNAREs) can form a stable SNARE complex to bridge and drive membrane fusion (Bock et al., 2001; Jahn and Scheller, 2006; Lipka et al., 2007).

During AM symbiosis, the formation of PAM requires the delivery of an enormous amount of membrane material to the proximity of branched fungal hyphae (Dickson and Kolesik, 1999; Harrison and Ivanov, 2017). Several specific SNAREs have been reported to be involved in this process. Two VAMP72 (vesicle-associated membrane protein 72) subfamily R-SNAREs, VAMP721d and VAMP721e in *Medicago truncatula*, were first revealed to be involved in the formation of the plant-fungus interface (Ivanov et al., 2012). Simultaneously silencing *MtVAMP721d* and *MtVAMP721e* by RNA interference (RNAi) led to a marked decrease of mature arbuscules and the accumulation of many abnormal arbuscules lacking fine-branches (Ivanov et al., 2012). Also, *Lotus japonicus VTI12*, encoding a Qb-SNARE, showed enhanced expression in mycorrhizal roots, and down-regulation of *LjVTI12* resulted in small and distorted arbuscules (Lota et al., 2013). Moreover, a SYNTAXIN OF PLANTS 13 subfamily clade II (SYP13II) member, *SYP132* encoding a Qa-SNARE in *M. truncatula*, is able to produce two isoforms, *SYP132*α and *SYP132*β via alternative cleavage and polyadenylation (APA). Relative to *SYP132*β, *SYP132*α exhibited higher expression in the arbuscule-containing cells. Silencing *SYP132*α caused many underdeveloped arbuscules, while silencing *SYP132*β seemed to inhibit root growth, thus indicating functional divergences of the two isoforms (Huisman et al., 2016; Pan et al., 2016).

Phylogenetic analyses showed that both *MtVAMP721d/e* and *MtSYP132* belong to the AM host-specific ‘symbiotic’ clades that lack representative genes from many non-mycorrhizal species, such as *Arabidopsis thaliana, Striga hermonthica* and *Beta vulgaris* (Ivanov et al., 2012; Huisman et al., 2016; Pan et al., 2016). Since *MtVAMP721d/e* and *MtSYP132*α RNAi roots showed similar phenotypes of abnormal arbuscules, it was hypothesized that MtSYP132α can interact with MtVAMP721d/e to define a symbiosis-specific membrane trafficking pathway, which is used to deliver specific cargo to the plant-fungus interface (Huisman et al., 2016; Pan et al., 2016). However, investigating more *Qa-SNARE* genes of SYP11/12/13 subfamilies and *R-SNARE* genes of VAMP72 subfamily in *M. truncatula* revealed a rather unspecific pattern. When driven by the promoter of *Medicago PT4 (Phosphate transporter 4*), subcellular localization to the PAM were observed not only for symbiotic *SNAREs*, but also for non-symbiotic *SNAREs* such as *SYP121*, *SYP131*, *SYP132*β, *VAMP721a* and *VAMP724* (Huisman et al., 2020). Also, non-symbiotic *SNAREs* can restore the arbuscule phenotypes in *MtSYP132*α or *MtVAMP712d/e* RNAi roots, if they were under the control of *MtSYP132* or *MtVAMP721e* promoters (Huisman et al., 2020). It remains to be explored whether non-symbiotic *SNAREs* are involved in symbiosis if their own promoters were maintained.

To gain more understanding on the functional divergences of different SNAREs, in this study, we investigated three *SYP13* genes in rice, including two genes, *OsSYP131a* and *OsSYP131b* in the non-symbiotic SYP13I clade and one gene *OsSYP132* in the symbiotic SYP13II clade. By generating mutants for each of these genes via CRISPR/Cas9 technique and obtaining the *OsSYP131b OsSYP132* double mutants, the potential roles of these Qa-SNARE-encoding genes in AM symbiosis and also in rice seed fertility were investigated. We discovered that *OsSYP131b* and *OsSYP132* single mutants displayed apparently normal mycorrhization patterns, but AM symbiosis is markedly disrupted by removing *OsSYP131b* and *OsSYP132* simultaneously. Our results suggest that both the constitutively expressed SYP13I clade and the symbiotically induced SYP13II clade of SYNTAXINs are involved in AM symbiosis.

## Materials and Methods

### Reconstructing a Phylogeny of Plant SYP13 Subfamily

The protein sequences of AtSYP131 (At3G03800), AtSYP132 (At5G08080) and MtSYP132 (Medtr2g088700) were used as query sequences to run BLASTP searches against the proteomes of 29 representative seed plants, including 4 gymnosperms and 25 angiosperms (for species list and genome information, see Supplementary Table 1). For each surveyed species, the obtained sequences showing an *E*-value < 10^–4^ and > 50% length coverage were kept and further examined for their resemblance with AtSYP131 rather than AtSYP111 (At1G08560) and AtSYP121 (At3G11820). A total of 58 sequences closely-related to the SYP13 subfamily were obtained from the surveyed species and aligned together with AtSYP111 and AtSYP121 (as outgroup) by ClustalW and Muscle programs, as implemented in MEGA v7.0 (Kumar et al., 2016). Among them, the rice gene *OsSYP132* (*LOC4343440* in NCBI database)^1^ was not annotated in the surveyed *O. sativa* ssp. japonica cv. Nipponbare genome (v7.0), but an identical gene (*OsKitaake07g168700*) was annotated in the *O. sativa* ssp. japonica cv. Kitaake genome at Phytozome v13^2^. After manual examination for site homology, the obtained alignment was used to reconstruct a maximum-likelihood tree of SYP13 subfamily under the GTR + F + I + G4 model via IQ-TREE, and the robustness of internal branches were examined by conducting the Shimodaira-Hasegawa approximate likelihood ratio test (SH-aLRT) and by performing 1,000 ultrafast bootstrap replicates (Guindon et al., 2010; Trifinopoulos et al., 2016).

### Plant Cultivation and Inoculation With *Rhizophagus irregularis*

The rice (*O. sativa* ssp. japonica) wild-type and transgenic plants used in this study were in the cv. Nipponbare background. Rice seeds were surface-sterilized with 2.5% sodium hypochlorite and 0.1% Tween20 for 15 min, 70% alcohol for 2 min, rinsed 3 times with sterile water, and germinated on a 1/2 modified Murashige-Skoog (MS) medium in a growth chamber with 12/12 h day/night cycle at 28°C/22°C and 70% relative humidity for two weeks. For AM colonization, seedlings were transplanted in pots (height 9 cm, diameter 12 cm) containing an autoclaved sand/perlite (3:1 [v/v]) mixture with three seedlings per pot. *Rhizophagus irregularis* fungal spores were freshly extracted from transgenic hairy carrot root cultures and were resuspended in water. Approximately 120 spores were added to the rhizosphere of each seedling. Plants were grown under a 16/8 h day/night cycle at 28°C/22°C and were fertilized twice a week with 1/2 Hoagland solution containing 20 μM phosphates. Plant roots were harvested at 3-week post inoculation (wpi) and 6-wpi, where half of the samples were used for staining and mycorrhizal phenotype examination and the other half were frozen in liquid nitrogen for RNA extraction.

For hydroponic culture, surface-sterilized rice seeds were germinated in sterile water for 2 days at 37°C, then transferred into hydroponic boxes (length 12.6 cm, width 8.5 cm, height 11 cm, 10 seedlings/box). The plants were grown in sterile water for one week, then in 1/4 Hoagland solution for another two weeks. To compare the root morphology between wild-type rice and the mutants, the aboveground height, primary root length (PRs), crown root (CRs) and lateral root numbers (LRs) were recorded each week.

### RNA Extraction, Transcriptome Sequencing, and Quantitative RT-PCR

The total RNA of rice roots was extracted by the Trizol method (Invitrogen, Carlsbad, CA, United States). The RNA purity and integrity were examined by RNA electrophoresis in 2.5% (wt/vol) agarose gel. RNA (1 μg) was treated with DNase I prior to cDNA synthesis using a Revert-Aid First Strand cDNA Synthesis Kit (Thermo Scientific). Quantitative RT-PCR (qRT-PCR) was performed by measuring the intensity of SYBR Green Fluorescent dye conjugated to double stranded DNA molecules using a C1000 Thermal Cycler Real-Time PCR detection system (Bio-Rad). Gene expression values were normalized to the rice housekeeping gene, *ubiquitin 1* (*OsUBI1*) (Chen et al., 2008), and analyzed by the 2^–Δ^ *^CT^* method (Livak and Schmittgen, 2001). Statistical differences were tested via the Student’s t-test (p < 0.05). All the qRT-PCR primers used in this study are provided in Supplementary Table 2.

### CRISPR Vector Construction and Obtaining *Ossyp13* Mutant Lines

To generate single mutants for three *OsSYP13* genes, the Clustered Regularly Interspaced Short Palindromic Repeats (CRISPR)/Cas9 technique was employed. Single-guide RNAs (sgRNAs) targeting *OsSYP131a*, *OsSYP131b* and *OsSYP132*, respectively, were designed using the CRISPR-PLANT^3^ and CRISPRdirect^4^ online tools (Xie et al., 2014; Naito et al., 2015). For each sgRNA, a pair of complementary primers with sticky ends were synthesized, annealed, and ligated with a linear CRISPR vector, pRGEB31, which was pre-digested by the restriction enzyme, *Bsa*I. After transforming rice calli via agrobacteria containing the pRGEB31-sgRNA vectors (Liu et al., 2022), the editing alleles of *OsSYP13* were examined through PCR and sequencing with specific primers, *OsSYP131a* edit-F/R, *OsSYP131b* edit-F/R and *OsSYP132* edit-F/R, respectively, which all covered the editing regions (Supplementary Figure 2 and Supplementary Table 2). In subsequent generations, plants carrying desired homozygous mutations without the pRGEB31 construct, which can be detected by *pRGEB31-Cas9*-F/R, were selected for reproduction (Supplementary Table 2). Finally, the T_2_ generations of the mutant lines were used for further studies.

*Ossyp131b Ossyp132* double mutant lines were obtained by hybridization between homozygous *Ossyp132-2* and *Ossyp131b-1* or *Ossyp131b-2* single mutants. The doubly heterozygous F_1_ progeny were grown to maturity and allowed to self-pollinate, and seeds containing the segregating F_2_ embryos were obtained. Because the *OsSYP131b* and *OsSYP132* genes are located on separate chromosomes and are hence genetically unlinked, in such a cross, we would expect to find a 15: 1 ratio of F_2_ progeny with other genotypes to that of the *Ossyp131b Ossyp132* doubly homozygous mutant. The doubly homozygous F_2_ progeny were selected for reproduction and the obtained F_3_ generation mutants were used for further studies.

### Fungal Structure Staining, Quantification and Microscopic Imaging

For wheat germ agglutinin (WGA) staining (Schaffer and Peterson, 1993), fresh rice roots were fixed in 50% ethanol for > 4 h and cleared in 20% KOH (w/v) for 2–3 d at room temperature. Next, the roots were acidified in 0.1 M HCl for 20 min and rinsed 5 times with phosphate-buffered saline (PBS) solution (pH 7.4). After incubating overnight in PBS solution containing 1 μg/mL WGA-Alexa Fluor 488 (Invitrogen) in the dark and washing with PBS, the stained roots were examined under a confocal Olympus FV10-ASM microscope (Olympus, Tokyo, Japan) and representative images were captured. For ink-vinegar staining (Vierheilig et al., 1998), rice roots were cleared with 20% KOH for 40 to 120 min at 65°C, acidified by 5% acetic acid for 5 min at room temperature, incubated in ink-vinegar solution for 30 min at 65°C, and decolored in tap water for 14 h. The stained roots were examined by a Nikon E100 light microscope (Nikon, Tokyo, Japan). To quantify the fungal colonization rate, a modified gridline intersect method (McGonigle et al., 1990) was used to observe and record fungal structures, including hyphopodia, intraradical hyphae, arbuscules, and vesicles. For arbuscule size measurement, after ink-vinegar staining, the images of colonized root fragments were taken by JieDa microscopic imaging software and the longitudinal lengths of arbuscules were measured by using a ruler implemented in the software. For either wild-type rice or the mutants, at least 15 root fragments from three individuals were measured at 3- and 6-wpi. The sizes of arbuscules were determined by measuring the length of all visible arbuscules in independent infection units (from hyphopodia to the running edge of hyphae toward the root tip) and classified into eight clusters from 0 to 10 μm to > 70 μm. The numbers in different clusters were divided by the number of all measured arbuscules from each genotype to get the percentage of arbuscule size classes.

## Results

### The Rice SYP13 Subfamily Qa-SNAREs Include Two SYP13I Members and One SYP13II Member

**FIGURE 1:**
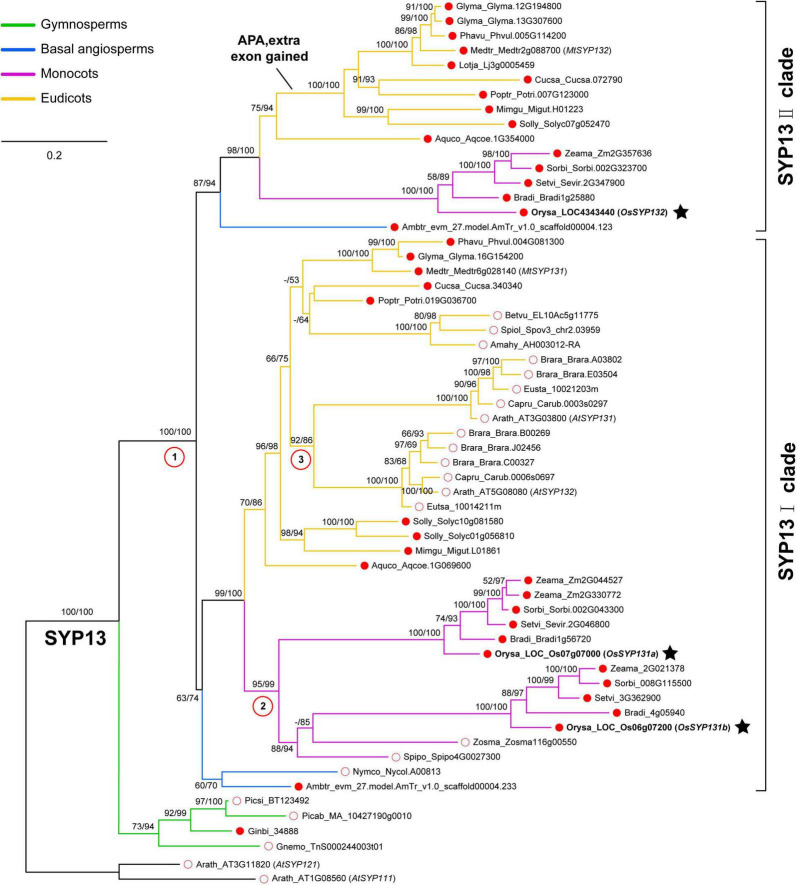
Phylogenetic analyses of SYNTAXIN OF PLANTS 13 (SYP13) subfamily including 29 seed plants. The phylogeny was reconstructed using a maximum-likelihood method under the GTR + F + I + G4 model via IQ-TREE. Branch support values were examined by conducting the Shimodaira-Hasegawa approximate likelihood ratio test (SH-aLRT) and by performing 1,000 ultrafast bootstrap replicates. Two *Arabidopsis thaliana SYP1* genes (*AtSYP111* and *AtSYP121*) were used as the outgroup. There were three duplication events in angiosperms marked by red circles with numbers. The ancestral *SYP13* gene was first duplicated into two clades, SYP13I and SYP13II; then two independent duplications occurred in the common ancestor of Brassicaceae or that of monocots, respectively. Three rice *SYP13* genes identified in this work were indicated by black stars. Red dot and red circle indicate AMS host plants and AMS non-host plants, respectively. The sequences from gymnosperms, basal angiosperm, monocots and eudicots were showed in different colors.

After identifying a total of 58 SYP13 subfamily members from 29 surveyed seed plants (Supplementary Table 1), a maximum-likelihood (ML) tree was reconstructed (see Materials and Methods). One *SYP13* gene was found in each of the four surveyed gymnosperms and these sequences formed a monophyletic group sister to all the angiosperm *SYP13* sequences, suggesting that there was one ancestral *SYP13* gene in the common ancestor of seed plants (Figure 1). In angiosperms, however, the ancestral gene had further duplicated into two lineages, namely SYP13I and SYP13II clades (Figure 1).

Within the SYP13I clade, representative genes were found in both mycorrhizal and non-mycorrhizal species. The two basal angiosperm species *Amborella trichopoda* and *Nymphaea colorata* and many dicot species have all maintained one member in this clade (Figure 1). However, two independent duplication events likely occurred in the common ancestor of Brassicaceae species and in the common ancestor of monocots, respectively. As a result, *A. thaliana* possesses two *SYP13I* genes, which were previously named as *AtSYP131* and *AtSYP132*, and their encoded proteins share ∼71% identities. Similarly, two *SYP13I* genes were present in the rice (*Oryza sativa*) genome. In this study, we referred to gene *Os07g07000* as *OsSYP131a* and gene *Os06g07200* as *OsSYP131b* (Figure 1). Their protein sequences share ∼61% identities.

Within the SYP13II clade, representative genes were not detected in the 10 surveyed non-mycorrhizal angiosperms, including the aquatic basal angiosperm *N. colorata*, two aquatic monocot species *Spirodela polyrhiza* and *Zostera marina*, three Amaranthaceae species and four Brassicaceae species (Supplementary Table 1). The remaining 15 mycorrhizal angiosperms all possess one *SYP13II* gene, except soybean (*Glycine max*), with two copies. An extra exon 14 of *SYP13II* could be found in all the mycorrhizal dicot species except *A. coerulea*, suggesting that these dicot species can produce two isoforms of *SYP13II* gene via APA as in *M. truncatula*. Differently, the basal angiosperm *A. trichopoda*, the five mycorrhizal monocot species and the basal eudicot species *A. coerulea* all possess a *SYP13II* gene without the extra exon. In rice, one gene *OsSYP132* (*LOC4343440*) belongs to this clade and it encodes a protein sharing ∼58% sequence identities to OsSYP131a and ∼55% identities to OsSYP131b.

### *OsSYP132*, but Not *OsSYP131a* or *OsSYP131b*, Is Induced in Rice Roots During AM Symbiosis

A previous research provided a detailed transcriptome data for various rice organs (roots, leaves, flowers and seeds) at different developmental stages (Wang et al., 2015). According to this study, *OsSYP132* was barely expressed in leaves, flowers, seeds and was expressed in roots at a low level before flowering, while *OsSYP131a* was expressed in most tissues including flowers, and *OsSYP131b* was expressed at a rather high level in roots and leaves (Supplementary Figure 1).

**FIGURE 2:**
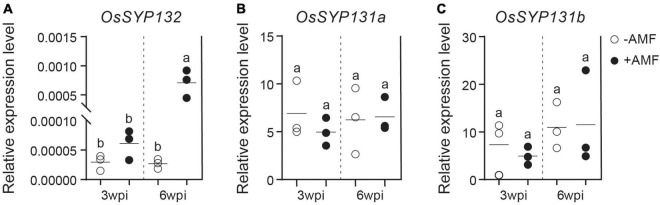
The expression patterns of three *OsSYP13* genes in rice roots during arbuscular mycorrhizal (AM) symbiosis **(A–C)**. The relative expression levels of *OsSYP132*, *OsSYP131a* and *OsSYP131b* were measured by Quantitative RT-PCR (qRT-PCR). Rice wild-type (WT) roots were either mock inoculated (–AMF) or colonized by *Rhizophagus irregularis* (+ AMF) were harvested at 3- and 6-weeks post-inoculation (wpi). The gene expression values were normalized to the rice housekeeping gene, *ubiquitin 1* (*OsUBI1*). Data are individual values from three biological replicates and the horizontal line shows the mean value. Different letters indicate significant statistical differences between groups evaluated by the Tukey’s honest significance test (between the pairs, *P* < 0.05 or *P* > 0.05). This is a representative experiment that was repeated three times.

To explore the expression patterns of *OsSYP13* genes during AM symbiosis, the root samples of rice individuals inoculated by the AM fungus (AMF) *Rhizophagus irregularis* or by mock-solution were respectively collected at two different times points, 3- and 6-weeks post-inoculation (wpi). Quantitative RT-PCR (qRT-PCR) experiments were further conducted. At 3-wpi, the relative expression level of *OsSYP132* in mycorrhizal roots was only slightly higher than in control roots (Figure 2A, *p* > 0.05), while at 6-wpi, it became ∼25-fold higher than control (Figure 2A, *p* < 0.02). On the contrary, for *OsSYP131a* or *OsSYP131b*, similar relative expression levels were observed between mycorrhizal and non-mycorrhizal roots at both 3- and 6-wpi (Figures 2B,C).

### CRISPR/Cas9-Mediated Mutagenesis of *OsSYP13* Genes

To explore the potential roles of these three *OsSYP13* genes in AM symbiosis and/or in plant growth and development, we tried to obtain their null mutants using CRISPR/Cas9 technique (see Materials and Methods). For each gene, four to six mutated alleles were detected in the T_0_ generation (Supplementary Figure 2). Two alleles per gene were then chosen to obtain their homozygotes in subsequent generations, meanwhile eliminating the pRGEB31 vector. By introducing a 1-nucleotide deletion in exon 6, *Ossyp132-1* allele showed a premature stop codon, thus encoding a truncated protein only 123 aa in length (Supplementary Figure 2A). Similarly, the *Ossyp132-2* allele had a 1-nucleotide deletion in exon 8 and also produced a premature stop codon, encoding a truncated protein of 169 aa (Supplementary Figure 2A). Due to different 1-nucleotide insertions in exon 8, both *Ossyp131a-1* and *Ossyp131a-2* alleles had altered reading frame, consequently encoding truncated proteins only 155 aa in length (Supplementary Figure 2B). As for the *OsSYP131b* gene, *Ossyp131b-1* had a 1-nucleotide deletion and *Ossyp131b-2* showed a 1-nucleotide insertion in exon 6. Premature stop codons were produced, consequently encoding truncated proteins of 136 aa and 121 aa, respectively (Supplementary Figure 2C). Since all these mutated alleles resulted in truncated proteins that lack both SNARE domain and the transmembrane domain, it can be expected that these mutated alleles would lose the functions of *OsSYP13* genes.

To preclude off-target effects in CRISPR/Cas9-mediated genome editing, we further examined the relative expression levels of three *OsSYP13* genes in the *Ossyp131b* or *Ossyp132* mutants by qRT-PCR at 6 wpi by *R. irregularis*. The results showed that although the mutated gene was expressed at markedly reduced levels, the relative expression levels of the other two homologous genes were not affected (Supplementary Figure 3).

### The *Ossyp131a* T_0_ Homozygote Mutants Showed Defects in Seed Fertility

**FIGURE 3:**
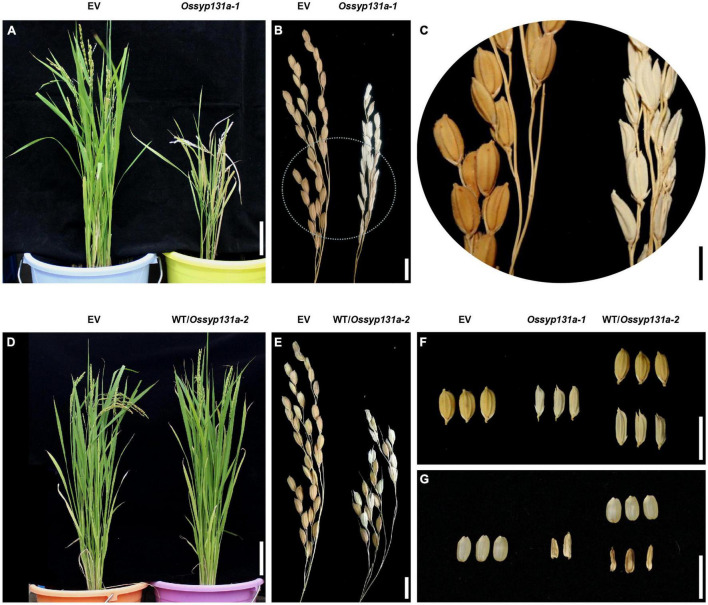
The *Ossyp131a* T_0_ mutants showed defects in growth and seed fertility **(A–C)**. The growth phenotype **(A)** and mature panicles **(B)** of a control T_0_ rice plant (with an empty vector, EV) and a *Ossyp131a-1* T_0_ homozygous mutant; **(C)** An amplified circle region in **(B)**; **(D,E)**, The growth phenotype **(D)** and mature panicles **(E)** of a control T_0_ rice plant (EV) and a *Ossyp131a-2* T_0_ heterozygous mutant; **(F–G)** The rice grain morphology of control T_0_ rice plant (EV), the *Ossyp131a-1* T_0_ homozygous mutant and the *Ossyp131a-2* T_0_ heterozygous mutant, either with husks **(F)** or without husks **(G)**. Scale bars = 10 cm **(A,D)**, 1 cm **(B,E–G)** or 0.5 cm **(C)**.

In the T_0_ generation, compared to the control plants transformed with an empty vector (EV), the *Ossyp131a-1* homozygous mutant exhibited a dwarf phenotype (Figure 3A). Moreover, unlike the healthy grains seen on control plants, only shriveled infertile grains were produced on the *Ossyp131a-1* T_0_ homozygous mutant, indicating a required role of *OsSYP13a* in rice seed fertility (Figures 3B,C). A heterozygous T_0_ plant (WT/*Ossyp131a-2*), instead, showed normal vegetative growth phenotype, and produced both healthy and infertile grains (Figures 3D-G). Germinating the produced healthy seeds and further examining their genotypes via sequencing then revealed a 1:2 ratio of WT to WT/*Ossyp131a-2* heterozygotes at the T_1_ generation (n = 56, 19: 37, χ2 = 0.009, P > 0.05), indicating that the *Ossyp131a-2* homozygous grains were also infertile.

### Neither the *Ossyp131b* nor *Ossyp132* Mutants Showed Defective AM Phenotype

After obtaining the homozygous *Ossyp131b* and *Ossyp132* mutants in the T_2_ generation, we first performed hydroponic cultivation experiments to examine their growth phenotypes under asymbiotic condition (see Materials and Methods). After 3-weeks cultivation, the *Ossyp131b* and *Ossyp132* mutants all showed similar values to wild-type rice in terms of above-ground height, maximum length of primary root, number of crown roots and number of lateral roots (Supplementary Figure 4), indicating a normal growth phenotype of these mutants under our experimental condition.

**FIGURE 4:**
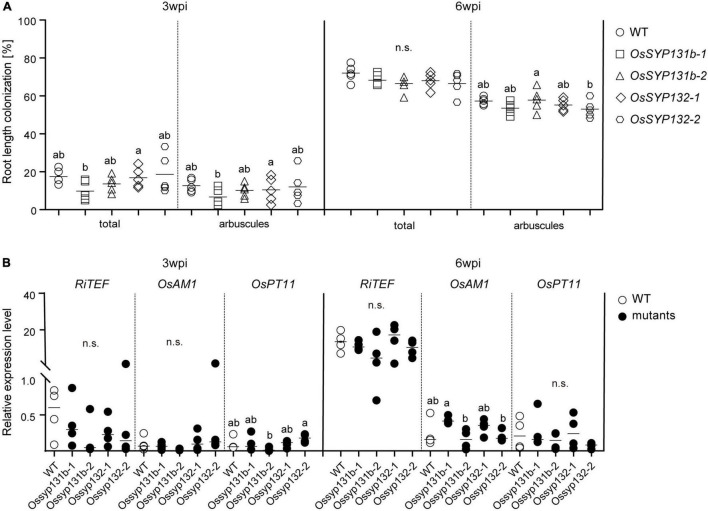
AM symbiosis was normal in the *Ossyp131b* and *Ossyp132* single mutants. **(A)** The total root length colonization rates and arbuscule abundance (%) of WT rice and *Ossyp131b*, *Ossyp132* mutants at 3- and 6-wpi by *R. irregularis* were determined by the gridline intersect method. Data were individual values from five biological replicates and the horizontal line showed the mean value. Different letters indicate statistical differences between genotypes evaluated by the Tukey’s honest significance test (all pairs between WT and mutants, *P* > 0.05, n.s., no significance). **(B)** Transcript accumulation of *OsRiTEF* (*Translation Elongation Factor*), *OsAM1* and *OsPT11* (*Phosphate transporter 11*) were measured by qRT-PCR in WT roots and mutants in the presence of *R. irregularis* at 3- and 6-wpi. The housekeeping gene, *OsUBI1*, was used for normalization. Data were individual values from four biological replicates and the horizontal line showed the mean value. Different letters indicate statistical differences evaluated by the Tukey’s test, too (all pairs between WT and mutants, *P* > 0.05, n.s., no significance). This is a representative experiment that was repeated three times.

We then examined the mycorrhizal phenotypes of the *Ossyp131b* and *Ossyp132* mutants. At 3-wpi by *R. irregularis*, an average of ∼18% wild-type rice lateral roots was colonized, including ∼13% root length forming arbuscules (Figure 4A). Rather surprisingly, comparable total colonization levels (10-19%) and arbuscule abundance (7-12%) were observed in the examined *Ossyp131b* and *Ossyp132* mutants, with arbuscular morphology unaffected (Figure 4A and Supplementary Figure 5). At 6-wpi, the total colonization levels still showed no statistically significant differences between wild-type and the mutants (∼72% vs. 67-68%). Neither did the arbuscule abundance levels, which was ∼57% in WT and 53-58% in the mutants (Figure 4A).

The unaffected AMF colonization phenotype in the *Ossyp131b* and *Ossyp132* mutants was then confirmed by measuring the relative expression levels of three AM marker genes, *RiTEF* (*Translation Elongation Factor*), *OsAM1* and *OsPT11* (*Phosphate transporter 11*). The fungal housekeeping gene *RiTEF* represents a molecular marker correlated with the abundance of intraradical fungal structures, while locating to the PAM. *OsAM1* is early expressed in rice cells colonized by small arbuscules and in cortex cells flanking intercellularly growing hyphae. *OsPT11* is mainly responsible for transferring phosphate from fungi to plants. For all the three marker genes, comparable relative expression levels were detected between wild-type rice and the examined mutants, thus supporting that AM symbiosis was successfully established in the *Ossyp131b* and *Ossyp132* mutants as in wild-type (Figure 4B).

### Loss of Functions of Both *OsSYP131b* and *OsSYP132* Strongly Inhibited AMF Colonization in Rice Roots

To further test whether *OsSYP131b* and *OsSYP132* play redundant roles in AM symbiosis, a homozygous *Ossyp132-2* mutant was crossed with the *Ossyp131b-1* or *Ossyp131b-2* mutants, respectively. The double mutants were identified from F_2_ population and further generated homozygous *Ossyp131b-1 Ossyp132-2* and *Ossyp131b-2 Ossyp132-2* double mutants at F_3_ generation. Hydroponic cultivation experiments were also performed for the double mutants, which showed normal growth phenotypes compared to wild-type (Supplementary Figure 4).

**FIGURE 5:**
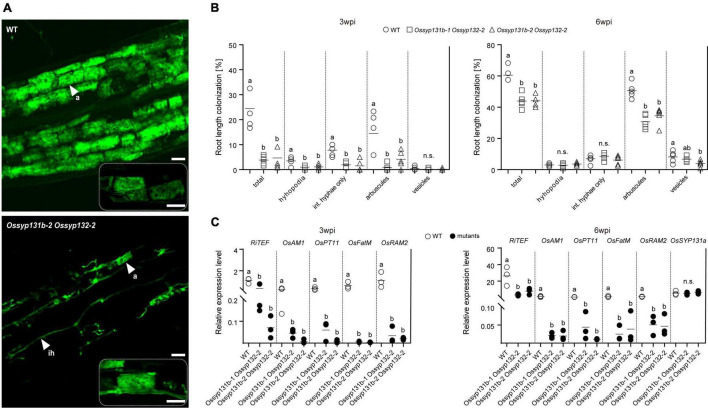
AM fungal colonization was impaired in the *Ossyp131b Ossyp132* double mutants. **(A)** Laser scanning confocal images showing fungal structures stained by WGA-Alexa Fluor 488 in WT rice roots and the *Ossyp131b Ossyp132* double mutants colonized by *R. irregularis* at 6-wpi. White arrows indicate mycorrhizal structures: a, arbuscule; ih, intraradical hyphae. Scale bars = 20 μm (above images), 50 μm (below images), or 25 μm (rectangle images with dotted lines). **(B)** The root length colonization rates (%) containing total, hyphopodia, intraradical hyphae, arbuscules, and vesicles of WT rice and the *Ossyp131b Ossyp132* double mutants by *R. irregularis* at 3- and 6-wpi were determined by the gridline intersect method. Data were individual values from five biological replicates and the horizontal line showed the mean value. Different letters indicate significant statistical differences between genotypes evaluated by the Tukey’s honest significance test (between the pairs, *P* < 0.05 or *P* > 0.05, n.s., no significance). Int. hyphae, intraradical hyphae. **(C)** The transcript accumulation of AM marker genes *RiTEF*, *OsAM1*, *OsPT11*, *OsFatM* (*fat required for AM symbiosis*) and *OsRAM2* (*required for arbuscular mycorrhization2*) as well as *OsSYP131a* were measured by qRT-PCR in the WT roots and *Ossyp131b Ossyp132* double mutants by *R. irregularis*. The housekeeping gene, *OsUBI1*, was used for normalization. Data are individual values from three biological replicates and the horizontal line shows the mean value. Different letters indicate levels of statistical differences evaluated by the Tukey’s test, too (all pairs except the *OsSYP131a* gene, *P* < 0.05). This is a representative experiment that was repeated three times with similar results.

We then assessed the mycorrhizal phenotypes. This time, the total colonization rates by AMF in the *Ossyp131b Ossyp132* double mutants were only 4∼5% at 3-wpi, which was much lower than the average rate observed in wild-type (∼25%, *p* < 0.05). The arbuscule abundance level also greatly decreased from ∼14% in wild-type to only 1-4% in the double mutants (*p* < 0.05, Figures 5A,B), indicating that both fungal colonization and arbuscule formation were greatly delayed in the double mutants at early inoculation process. At 6-wpi, the AMF colonization rate in the double mutants increased to 43-44%, which was still much lower than in wild-type (∼61%, *p* < 0.05). Similarly, fewer arbuscules were observed in the double mutants comparing to wild-type (31-35% vs. 51%, *p* < 0.05). However, the mature arbuscules observed in the double mutants can occupy the colonized cells completely (Figure 5A). We then quantified and compared the arbuscular size distribution pattern between the double mutant and wild-type. As a result, no statistically significant differences on arbuscular size distribution were observed (Supplementary Figure 6), indicating that loss functions of both *OsSYP131b* and *OsSYP132* genes would not impair a specific stage of arbuscular development. Therefore, the rather fewer arbuscules and lower colonization rates observed in the double mutants were more likely due to a less efficient but still working membrane trafficking pathway, which affected fungal proliferation in rice roots.

Consistent with the impaired mycorrhizal phenotype, qRT-PCR results also demonstrated that the five marker genes *RiTEF*, *OsPT11, OsAM1*, *OsFatM* (*fat required for AM symbiosis*) and *OsRAM2* (*required for arbuscular mycorrhization2*) (Wang et al., 2012; Bravo et al., 2017; Jiang et al., 2017; Dai et al., 2022; Liu et al., 2022) were expressed at markedly reduced levels in the *Ossyp131b Ossyp132* double mutants than in WT plants at both examined time points (Figure 5C). The relative expression level of *OsSYP131a* gene was not affected in the double mutants, though (Figure 5C).

## Discussion

An interesting feature of AM symbiosis is that the fungal hyphae do not actually enter into the host cytoplasm, but are closely surrounded by host cell plasma membrane. As fungal hyphae continuously branch to form arbuscules in the inner cortical cells, the PAM also expands quickly (up to 10-fold) in order to completely surround the hyphal branches (Alexander et al., 1989). Multiple SNARE proteins likely involved in PAM formation have been identified in legume species, including *MtVAMP721d* and *MtVAMP721e* from a ‘symbiotic’ VAMP72II clade and *MtSYP132* from a ‘symbiotic’ SYP13II clade (Ivanov et al., 2012; Huisman et al., 2016, 2020; Pan et al., 2016). It was wondered whether functional divergences could have occurred for SNAREs of the ‘symbiotic’ clades, consequently defining a symbiosis-specific membrane trafficking pathway.

To explore this question, we reconstructed a phylogeny of plant SYP13 subfamily (Figure 1). It illustrated that there was an ancestral *SYP13* gene in the common ancestor of seed plants, which further duplicated into two lineages in angiosperms, namely the ‘non-symbiotic’ SYP13I clade (including both mycorrhizal and non-mycorrhizal host species) and the ‘symbiotic’ SYP13II clade that includes only mycorrhizal species (Figure 1). Within the SYP13I clade, two independent duplication events had occurred in the common ancestor of monocots and in the common ancestor of Brassicaceae, respectively (Figure 1). Moreover, an unequal crossover event likely occurred between homologous members of the two SYP13 clades in the dicot lineage after the split of *A. coerulea* (Huisman et al., 2016), resulting in chimeric *SYP13II* genes that can produce two isoforms in most dicot species. These evolutionary events had complicated gene relationships among different plant species (especially in dicots), making it difficult to explore functional similarities or divergences among genes of the two SYP13 clades.

Having lost the representative gene from the SYP13II clade, *A. thaliana* has two representative genes, *AtSYP131* and *AtSYP132* in the SYP13I clade. Their encoded proteins were both revealed to be located on the plasma membrane, like other Qa-SNAREs in SYP11 and SYP12 subfamilies (Uemura et al., 2004). *AtSYP131* was specifically expressed in mature pollen grains, while *AtSYP132* exhibit a ubiquitous expression pattern in all tissues at all developmental stages (Enami et al., 2009). A T-DNA insertion in the promoter region of *AtSYP132* resulted in bushy plants with almost no seeds, and the seedlings also formed adventitious roots instead of a single primary root, demonstrating that *AtSYP132* likely plays essential roles in plant growth and development (Park et al., 2018). Downregulating *AtSYP132* via artificial microRNA also revealed that this gene plays an important role in cytokinesis (Park et al., 2018).

In the legume *M. truncatula*, gene *MtSYP131* belongs to the SYP13I clade, while gene *MtSYP132* of the SYP13II clade can produce two isoforms *MtSYP132*α and *MtSYP132*β. The function of *MtSYP131* is not known yet, although this gene was found expressed in arbusculated cells together with other SYP11 and SYP12 members (Huisman et al., 2020). Silencing *MtSYP132*α caused underdeveloped arbuscule phenotype, suggesting a specific role of this isoform in symbiosis; meanwhile, silencing *MtSYP132*β largely inhibited root growth, which is analogous to the lethal phenotype of *AtSYP132* (Huisman et al., 2016; Pan et al., 2016). It should be noticed, however, silencing *MtSYP132*α seemed not only decreased the transcript abundance levels of *MtSYP132*α, but also reduced *MtSYP132*β expression at least in one of the previous studies (Pan et al., 2016), making it unclear whether both isoforms are involved in AM symbiosis. Moreover, after deleting the alternatively spliced exon of *Mtsyp132*α, the constitutively spliced *MtSYP132*β (with elevated expression levels than in wild-type) can functionally replace *MtSYP132*α to restore normal arbuscule morphology (Huisman et al., 2020). It therefore seemed to suggest that both isoforms are involved in regulating AM symbiosis. However, direct genetic evidence is lacking since silencing *MtSYP132*β was lethal and inhibited root growth.

Without forming chimeric genes, the single copy of *SYP13II* gene in monocot species, such as rice *OsSYP132* serves as an ideal candidate to investigate whether it has evolved specific role in AM symbiosis. The results of qRT-PCR also showed that *OsSYP132* was barely expressed in control roots, but became strongly upregulated in mycorrhizal roots (Figure 2A). On the contrary, both *OsSYP131a* and *OsSYP131b* of the SYP13I clade were stably expressed in both the mock-inoculated and mycorrhizal rice roots (Figures 2B,C). Therefore, both phylogenetic and expression data suggested that *OsSYP132* likely have a specific role in AM symbiosis. However, our *OsSYP132* knockout revealed no apparent AM developmental and morphological defects (Figure 4A and Supplementary Figure 5), and it was further confirmed by the normal expressions of three AM marker genes (Figure 4B).

The strict evolutionary conservation of a symbiosis-specific form of *SYP132* is in sharp contrast to the lack of a significant AM symbiosis defect when this gene is removed. It therefore pointed to another possibility, that is, both *SYP13I* and *SYP13II* genes are involved in regulating AM symbiosis; meanwhile *SYP13I* genes are also involved in plant growth and development, so they are retained in non-mycorrhizal hosts. To verify this possibility, we further obtained the *Ossyp131b* mutants and the *Ossyp131b Ossyp132* double mutants. Although the *OsSYP131b* mutants showed no defective mycorrhizal phenotype (Figure 4 and Supplementary Figure 5), the loss of functions of both *OsSYP131b* and *OsSYP132* strongly inhibited AMF colonization rate and arbuscule abundance levels (Figure 5). This finding reveals that successful AM symbiosis employed SNARE proteins from not only SYP13II but also SYP13I clades. Possibly the active vesicle trafficking in arbuscular cells in higher plants need the combined coding capacity of multiple *SYP13* genes, whose protein products can all partner with symbiotically induced VAMP72 proteins. It remains to be seen whether a similar requirement exists in other species, especially dicots that employ the APA mechanism to produce a ‘symbiotic’ *SYP132* transcript.

As for *OsSYP131a*, our results showed that its T_0_ homozygous mutants exhibited a dwarf phenotype and were not able to develop fertile seeds (Figure 3), indicating a function of this gene in plant growth and in seed fertility, which is to some extents, analogous to the *AtSYP132* phenotype (Park et al., 2018). However, since no fertile mutant seeds could be obtained for this gene and further used to test the mycorrhizal phenotype, its potential involvement in AM symbiosis cannot be determined. It should be realized that the arbuscule morphology was normal and the AM colonization was not completely blocked in the *Ossyp131b Ossyp132* double mutants (Figure 5A and Supplementary Figure 6), implying that additional SNAREs encoded by either *OsSYP131a* or other syntaxin genes can still make membrane trafficking work in the colonized cells, though in a less efficient way.

In this study, we investigated three rice *SYP13* subfamily *Qa-SNARE* genes, *OsSYP131a*, *OsSYP131b* and *OsSYP132*. Among them, *OsSYP131a* is essential for rice seed embryogenesis; while *OsSYP131b* and *OsSYP132* are functionally redundant in regulating AM symbiosis, probably by mediating membrane trafficking to form the plant-fungus interface in the colonized cortical cells.

## Data Availability Statement

The datasets presented in this study can be found in online repositories. The names of the repository/repositories and accession number(s) can be found in the article/Supplementary Material.

## Author Contributions

BW and Y-NL designed the project. Y-NL and C-CL performed most of the experiments. RG, LT, J-FC, and Y-NW assisted in experiments. DW participated in the discussion and helped in writing. Y-NL and BW analyzed the data and wrote the manuscript. All authors read and approved the manuscript.

## Conflict of Interest

The authors declare that the research was conducted in the absence of any commercial or financial relationships that could be construed as a potential conflict of interest.

## Publisher’s Note

All claims expressed in this article are solely those of the authors and do not necessarily represent those of their affiliated organizations, or those of the publisher, the editors and the reviewers. Any product that may be evaluated in this article, or claim that may be made by its manufacturer, is not guaranteed or endorsed by the publisher.

## Footnotes

1
https://www.ncbi.nlm.nih.gov/

2
https://phytozome-next.jgi.doe.gov/

3
http://www.genome.arizona.edu/crispr

4
http://crispr.dbcls.jp
