# Supplementary material for: The Rice Qa-SNAREs in SYP13 Subfamily Are Involved in Regulating Arbuscular Mycorrhizal Symbiosis and Seed Fertility
Source: Front Plant Sci. 2022 May 18;13:898286. doi: 10.3389/fpls.2022.898286 (PMC9158536; doi:10.3389/fpls.2022.898286)
Supplement: Supplementary file 1 [file Table_1.docx]

**Table S1.** The *SYP13* genes identified from 29 surveyed plant genomes.

| **Organism** | **Version/**  **Reference** | **Website** | **Abbr** | **AM/NM** | **Gene** |
| --- | --- | --- | --- | --- | --- |
| *Picea sitchensis* | *[Q903_v1](https://www.ncbi.nlm.nih.gov/genome/13137?genome_assembly_id=766816)* | https://www.ncbi.nlm.nih.gov | Picsi | NM | BT123492 |
| *Picea abies* | Nystedt et al., Nature 2013 497:579-584 | https://www.ncbi.nlm.nih.gov/genome/11155?genome_assembly_id=367163 | Picab | NM | MA_10427190g0010 |
| *Ginkgo biloba* | Guan et al.,  Gigascience  2016, 5:496 | https://www.ncbi.nlm.nih.gov | Ginbi | AM | Gb_34888 |
| *Gnetum montanum* | Wan et al.,  Nature Plants 2018, 4:82–89 | https://datadryad.org | Gnemo | NM | TnS000244003t01 |
| *Amborella trichopoda* | *v1.0* | https://phytozome-next.jgi.doe.gov/ | Ambtr | AM | evm_27.model.AmTr_v1.0_scaffold00004.233  evm_27.model.AmTr_v1.0_scaffold00004.123 |
| *Nymphaea colorata* | *v1.2* | https://phytozome-next.jgi.doe.gov/ | Nymco | NM | Nycol.A00813 |
| *Spirodela polyrhiza* | *v2* | https://phytozome-next.jgi.doe.gov/ | Spipo | NM | Spipo4G0027300 |
| *Zostera marina* | *v2.2* | https://phytozome-next.jgi.doe.gov/ | Zosma | NM | Zosma116g00550 |
| *Brachypodium distachyon* | *v3.1* | https://phytozome-next.jgi.doe.gov/ | Bradi | AM | Bradi1g56720  Bradi4g05940  Bradi1g25880 |
| *Oryza sativa* | *v7.0* | https://phytozome-next.jgi.doe.gov/ | Orysa | AM | LOC_Os07g07000 (OsSYP131a)  LOC_Os06g07200 (OsSYP131b)  LOC4343440 (OsSYP132) |
| *Setaria viridis* | *v1.1* | https://phytozome-next.jgi.doe.gov/ | Setvi | AM | Sevir.3G362900  Sevir.2G046800  Sevir.2G347900 |
| *Zea mays* | *Ensembl-*  *18* | https://phytozome-next.jgi.doe.gov/ | Zeama | AM | Zm2G021378  Zm2G044527  Zm2G330772  Zm2G357636 |
| *Sorghum bicolor* | *v3.1.1* | https://phytozome-next.jgi.doe.gov/ | Sorbi | AM | Sorbi.008G115500  Sorbi.002G043300  Sorbi.002G323700 |
| *Aquilegia coerulea* | *v3.1* | https://phytozome-next.jgi.doe.gov/ | Aquco | AM | Aqcoe1G069600  Aqcoe1G354000 |
| *Solanum lycopersicum* | *iTAG2.4* | https://phytozome-next.jgi.doe.gov/ | Solly | AM | Solyc01g056810  Solyc10g081580  Solyc07g052470 |
| *Mimulus guttatus* | *v2.0* | https://phytozome-next.jgi.doe.gov/ | Mimgu | AM | Migut.L01861  Migut.H01223 |
| *Amaranthus hypochondriacus* | *v2.1* | https://phytozome-next.jgi.doe.gov/ | Amahy | NM | AH003012-RA |
| *Spinacia oleracea* | *Spov3* | https://phytozome-next.jgi.doe.gov/ | Spiol | NM | Spov3_chr2.03959 |
| *Beta vulgaris* | *EL10_1.0* | https://phytozome-next.jgi.doe.gov/ | Betvu | NM | EL10Ac5g11775 |
| *Eutrema salsugineum* | *v1.0* | https://phytozome-next.jgi.doe.gov/ | Eutsa | NM | 10014211m  10021203m |
| *Brassica rapa* | *FPsc v1.3* | https://phytozome-next.jgi.doe.gov/ | Brara | NM | Brara.A03802  Brara.E03504  Brara.C00327  Brara.B00269  Brara.J02456 |
| *Arabidopsis thaliana* | *TAIR10* | https://phytozome-next.jgi.doe.gov/ | Arath | NM | AT1G08560 (AtSYP111)  AT3G11820 (AtSYP121)  AT3G03800 (AtSYP131)  AT5G08080 (AtSYP132) |
| *Capsella rubella* | *v1.0* | https://phytozome-next.jgi.doe.gov/ | Capru | NM | Carub.0003s0297  Carub.0006s0697 |
| *Populus trichocarpa* | *v3.0* | https://phytozome-next.jgi.doe.gov/ | Poptr | AM | Potri.019G036700  Potri.007G123000 |
| *Cucumis sativus* | *v1.1* | https://phytozome-next.jgi.doe.gov/ | Cucsa | AM | Cucsa.072790  Cucsa.340340 |
| *Lotus japonicus* | *Lj1.0v1* | https://phytozome-next.jgi.doe.gov/ | Lotja | AM | Lj3g0005459 |
| *Medicago truncatula* | *Mt4.0v1* | https://phytozome-next.jgi.doe.gov/ | Medtr | AM | Medtr6g028140  Medtr2g088700 (MtSYP132) |
| *Phaseolus vulgaris* | *v2.1* | https://phytozome-next.jgi.doe.gov/ | Phavu | AM | Phvul.004G081300  Phvul.005G114200 |
| *Glycine max* | *Wm82.a2.v1* | https://phytozome-next.jgi.doe.gov/ | Glyma | AM | Glyma.16G154200  Glyma.12G194800  Glyma.13G307600 |
