# Supplementary material for: The Rice Qa-SNAREs in SYP13 Subfamily Are Involved in Regulating Arbuscular Mycorrhizal Symbiosis and Seed Fertility
Source: Front Plant Sci. 2022 May 18;13:898286. doi: 10.3389/fpls.2022.898286 (PMC9158536; doi:10.3389/fpls.2022.898286)
Supplement: Supplementary file 2 [file Table_2.DOCX]

**Table S2.** The primers used in this study.

| Primer names | Primer sequences (5’-3’) |
| --- | --- |
| For *OsSYP13* genes editing |  |
| *OsSYP131a*-spacer-F | ggcaCGACATCACGATACTCCTGC |
| *OsSYP131a*-spacer-R | aaacGCAGGAGTATCGTGATGTCG |
| *OsSYP131b*-spacer-F | ggcaAGAAAAACCAGGCTTCGGTA |
| *OsSYP131b*-spacer-R | aaacTACCGAAGCCTGGTTTTTCT |
| *OsSYP132*-spacer1-F | ggcaGCACAGGCGTAGACCGATCA |
| *OsSYP132*-spacer1-R | aaacTGATCGGTCTACGCCTGTGC |
| *OsSYP132*-spacer2-F | ggcaATTCAAGATGAGTACCGACA |
| *OsSYP132*-spacer2-R | aaacTGTCGGTACTCATCTTGAAT |
| *pRGEB31-Cas9*-F | CAGATGTGCAGTCAGGGACC |
| *pRGEB31-Cas9*-R | CATGCACGCGCTAAAAACGG |
| *OsSYP131a* edit-F | TGAAGTGGGGAAAATCGCTCG |
| *OsSYP131a* edit-R | GTGACGTTCCTGTATCTCTGC |
| *OsSYP131b* edit-F | AACTGCACGTCTGGCAAAAT |
| *OsSYP131b* edit-R | CAAGAGTAAGGCTATCAGTGCAA |
| *OsSYP132* edit-F | TGGGAAAATTACACGCACCA |
| *OsSYP132* edit-R | AGCACTACTGTCCTCACCTG |
|  |  |
| For qRT-PCR |  |
| *qOsUbi1*-F | TGCACCCTAGGGCTGTCAAC |
| *qOsUbi1*-R | GACGCTCTAGTTCTTGATCTTCTTC |
| *qOsPT11*-F | GAGAAGTTCCCTGCTTCAAGCA |
| *qOsPT11*-R | CATATCCCAGATGAGCGTATCATG |
| *qOsAM1*-F | ACCTCGCCAAAATATATGTATGCTATT |
| *qOsAM1*-R | TTTGCTTGCCACACGTTTTAA |
| *qOsRAM2*-F | ATGAACCCTAGCCCCGGAT |
| *qOsRAM2*-R | GATGTTGGGCTTGGACACGA |
| *qOsFatM*-F | GCTTGAGACTCTGCCTGACC |
| *qOsFatM*-R | CTCCCGGCATAATCGTGTCT |
| *qRiTEF*-F | TGTTGCTTTCGTCCCAATATC |
| *qRiTEF*-R | GGTTTATCGGTAGGTCGAG |
| *qOsSYP131a*-F | AAGCCTGGATGTGGGAAAGG |
| *qOsSYP131a*-R | ACGACATCACGATACTCCTGC |
| *qOsSYP131b*-F | TACCGGGATGTAGTGGAGCG |
| *qOsSYP131b*-R | GAGTGTCAAGCACCCTTCCA |
| *qOsSYP132*-F | GCGTTGCGAGGATGGTTTTC |
| *qOsSYP132*-R | ACCGTGGCTATTATCTGCCC |
