# Supplementary figures and images for: The Rice Qa-SNAREs in SYP13 Subfamily Are Involved in Regulating Arbuscular Mycorrhizal Symbiosis and Seed Fertility
Source: Front Plant Sci. 2022 May 18;13:898286. doi: 10.3389/fpls.2022.898286 (PMC9158536; doi:10.3389/fpls.2022.898286)

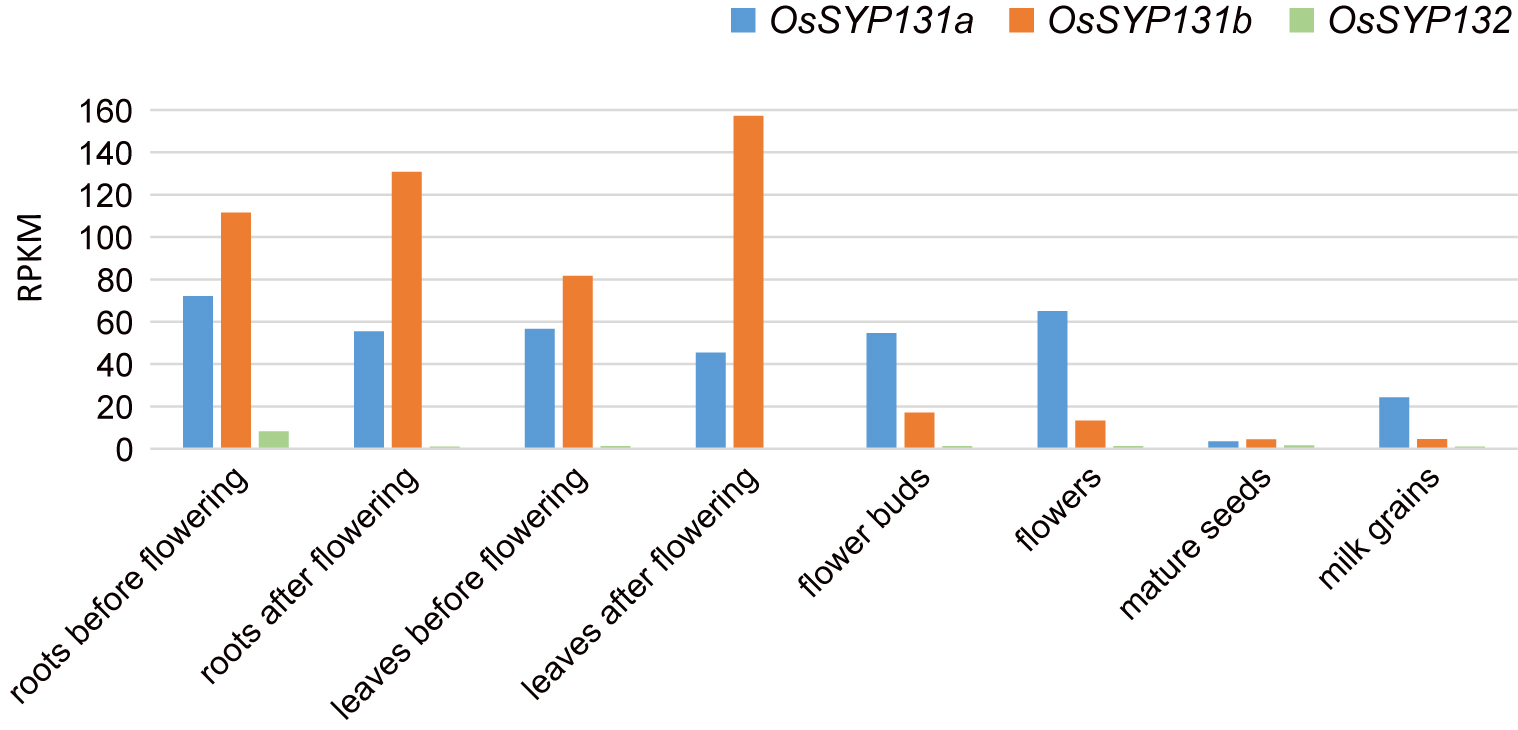

Supplement: Supplementary file 3 [file Image_1.JPEG]

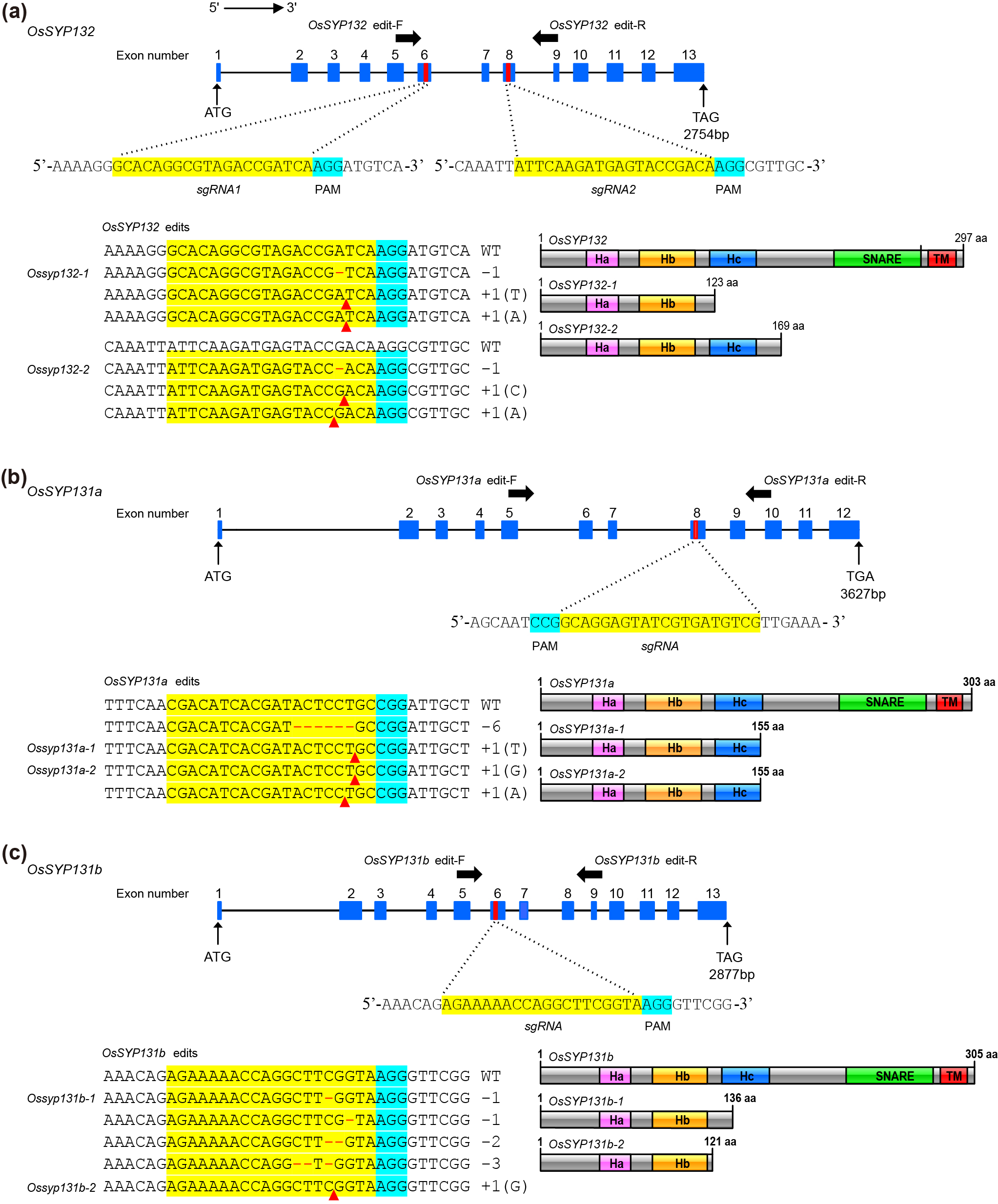

Supplement: Supplementary file 4 [file Image_2.JPEG]

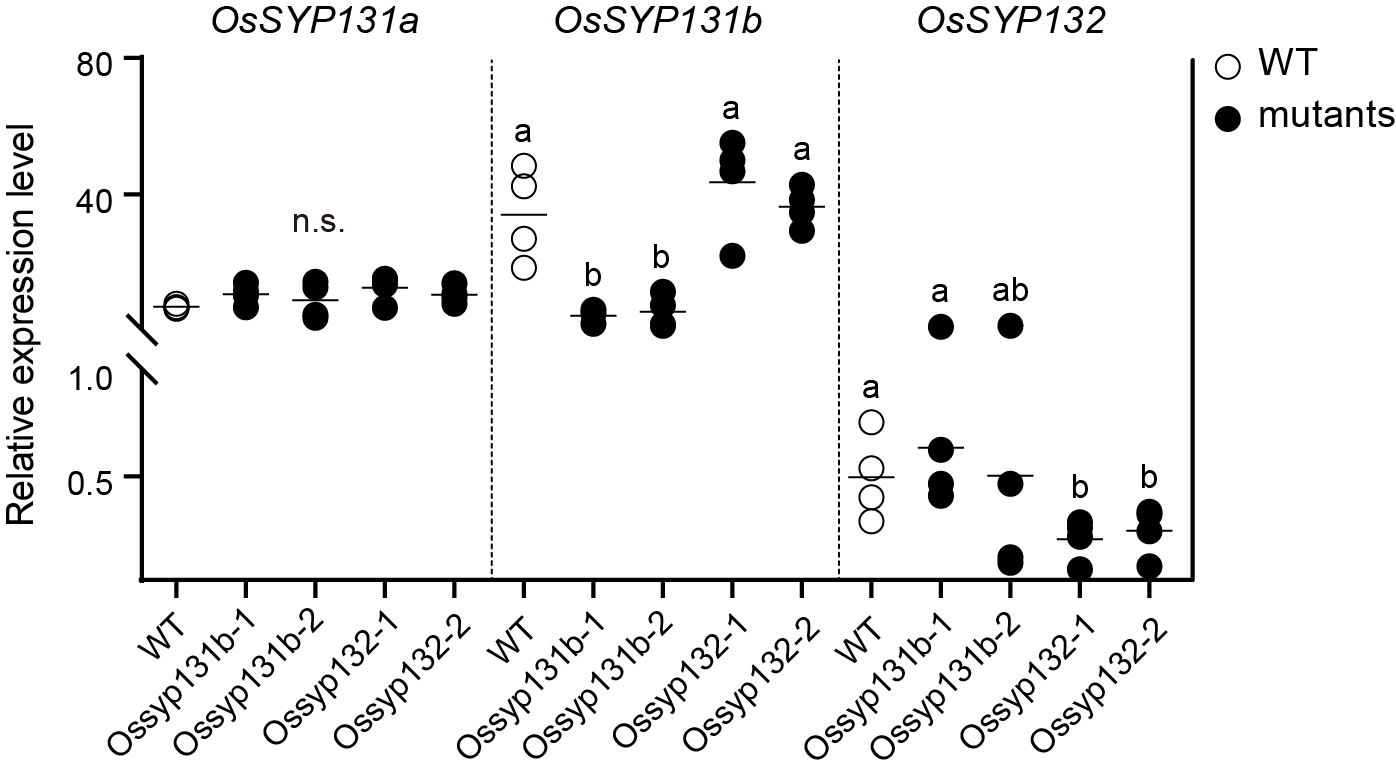

Supplement: Supplementary file 5 [file Image_3.JPEG]

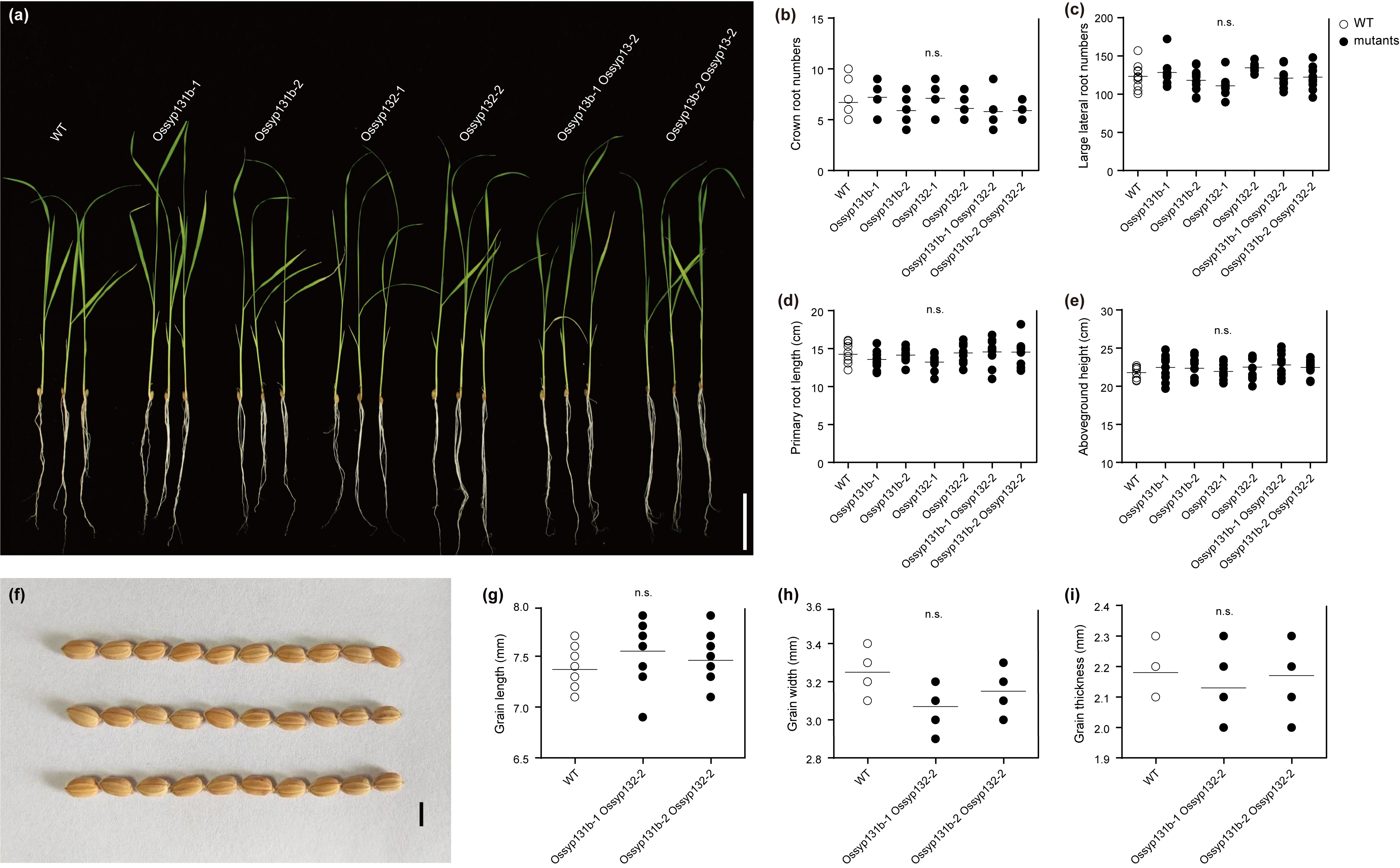

Supplement: Supplementary file 6 [file Image_4.JPEG]

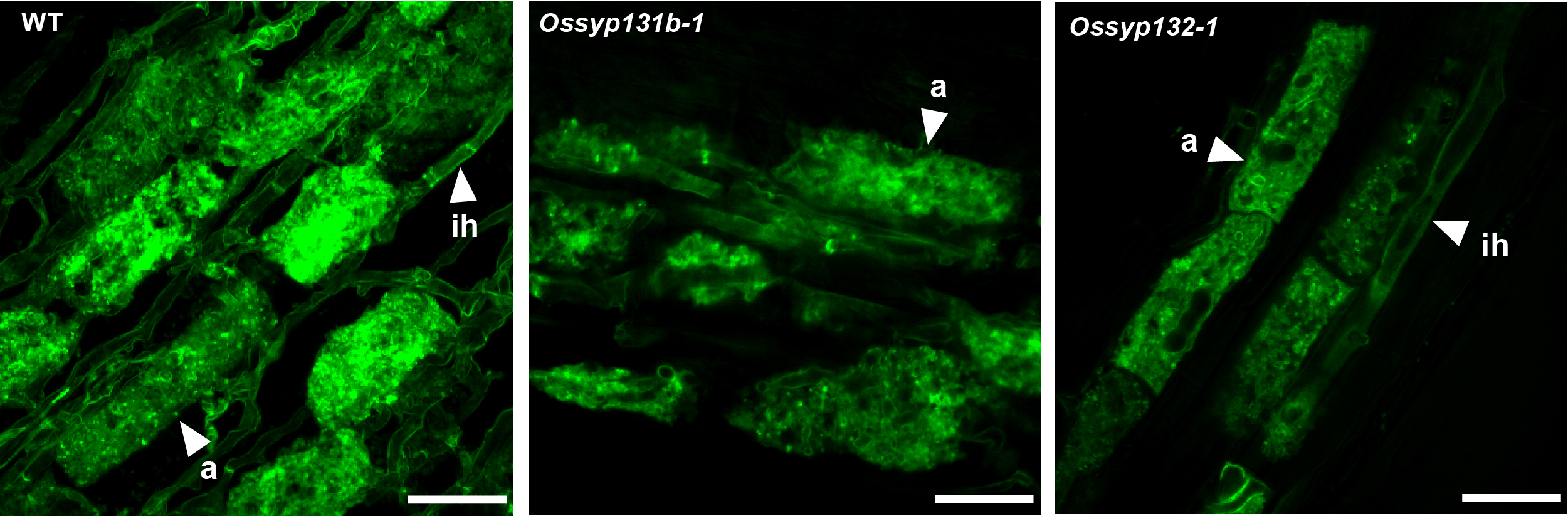

Supplement: Supplementary file 7 [file Image_5.JPEG]

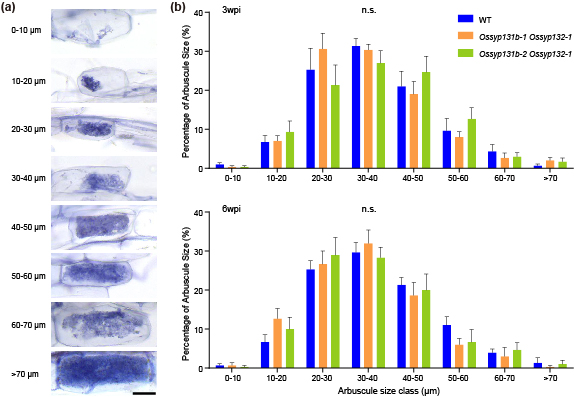

Supplement: Supplementary file 8 [file Image_6.JPEG]
